# Integrating plot-based methods for monitoring biodiversity in island habitats under the scope of BIODIVERSA+ project BioMonI: Beetle monitoring in Pico and Terceira Islands

**DOI:** 10.3897/BDJ.14.e189085

**Published:** 2026-04-24

**Authors:** Paulo A. V. Borges, Alexandra Dal Lago, Isabel R. Amorim, Rui Carvalho, Luís C. Crespo, Rui M.R. Nunes, Fernando Pereira, Carla Rego, Rosalina Gabriel, François Rigal, Pedro Cardoso

**Affiliations:** 1 University of the Azores, CE3C—Centre for Ecology, Evolution and Environmental Changes, Azorean Biodiversity Group, CHANGE —Global Change and Sustainability Institute, School of Agricultural and Environmental Sciences, Rua Capitão João d’Ávila, Pico da Urze, 9700-042, Angra do Heroísmo, Azores, Portugal University of the Azores, CE3C—Centre for Ecology, Evolution and Environmental Changes, Azorean Biodiversity Group, CHANGE —Global Change and Sustainability Institute, School of Agricultural and Environmental Sciences, Rua Capitão João d’Ávila, Pico da Urze, 9700-042 Angra do Heroísmo, Azores Portugal; 2 IUCN SSC Atlantic Islands Invertebrate Specialist Group, Angra do Heroísmo, Azores, Portugal IUCN SSC Atlantic Islands Invertebrate Specialist Group Angra do Heroísmo, Azores Portugal; 3 IUCN SSC Monitoring Specialist Group, Angra do Heroísmo, Azores, Portugal IUCN SSC Monitoring Specialist Group Angra do Heroísmo, Azores Portugal; 4 University of Bologna, Bologna, Italy University of Bologna Bologna Italy; 5 LIBRe – Laboratory for Integrative Biodiversity Research, Finnish Museum of Natural History, University of Helsinki, P.O.Box 17 (Pohjoinen Rautatiekatu 13), 00014, Helsinki, Finland LIBRe – Laboratory for Integrative Biodiversity Research, Finnish Museum of Natural History, University of Helsinki, P.O.Box 17 (Pohjoinen Rautatiekatu 13), 00014 Helsinki Finland; 6 Centre for Ecology, Evolution and Environmental Changes (CE3C) & Global Change and Sustainability Institute (CHANGE), Faculdade de Ciências, Universidade de Lisboa, Lisbon, Portugal Centre for Ecology, Evolution and Environmental Changes (CE3C) & Global Change and Sustainability Institute (CHANGE), Faculdade de Ciências, Universidade de Lisboa Lisbon Portugal; 7 Environment and Microbiology Team, Université de Pau et des Pays de l’Amour, Pau Cedex 64013, France Environment and Microbiology Team, Université de Pau et des Pays de l’Amour Pau Cedex 64013 France

**Keywords:** Azores Archipelago, native laurel forest, Coleoptera, COBRA protocol, long-term monitoring, Darwin Core

## Abstract

**Background:**

Oceanic island native forests have become highly fragmented and yet host a disproportionate share of endemic arthropod diversity. Long-term monitoring and conservation planning are often limited by the scarcity of standardised, plot-based datasets for key indicator taxonomic groups, such as the megadiverse beetles (Coleoptera). Under the scope of the projects EU-NETBIOME grant 0003/2011, FCT MACDIV – FCT-PTDC/BIABIC/0054/2014 and BIODIVERSA+ funded BioMonI, we compile and mobilise a baseline dataset of forest beetles from the Azores using a network of 16 permanent 50 m × 50 m native-forest plots (Pico: 6 plots; Terceira: 10 plots). Beetles were sampled with an optimised COBRA (Conservation Oriented Biodiversity Rapid Assessment) protocol complemented by beetle-targeted sampling techiques (under-bark/epiphyte and coarse woody debris/ground refugia searches), enabling repeatable and comparable monitoring across islands and through time.

**New information:**

We provide the first Darwin Core–compliant, plot-based inventory of beetles from native forests of Pico and Terceira Islands (Azores, Portugal), delivered as a sample-event dataset with 308 event records and an associated occurrence table (539 records). Across both islands, we recorded 43 beetle morphospecies (39 taxa were identified at species or subspecies levels) from 16 families, totalling 1,787 individuals. The plots in Pico yielded 25 taxa (13 families; 620 individuals; 23 identified species and subspecies) and those in Terceira 30 taxa (10 families; 1,167 individuals; 28 identified species and subspecies). The assemblage is dominated by endemic taxa in both richness and abundance, providing a robust benchmark for detecting compositional change, supporting biogeographical analyses and informing conservation assessments and management of native forest remnants, including evaluations of recovery status for threatened endemics.

## Introduction

Although six centuries of human settlement and land-use change have transformed the Azorean landscape, remnants of the original native laurel forest still persist (Elias et al. 2016). These forests currently cover only about 5% of the Archipelago and are mainly restricted to Flores, Pico and Terceira, forming small, isolated fragments embedded in a matrix of pastures, plantations and secondary vegetation (Norder et al. 2020). From a conservation perspective, these remnants are of outstanding importance because they host a high proportion of the Archipelago’s endemic arthropod fauna, many of which have already been assessed as threatened under the IUCN Red List framework (Borges et al. 2017b, Borges et al. 2018a). At the same time, numerous island arthropod species remain to be described or properly documented, despite substantial taxonomic and faunistic progress over the last decades (Borges et al. 2022).

Forest beetles (Insecta, Coleoptera) constitute a major component of the Azorean endemic diversity. They occupy a wide range of trophic and microhabitat niches as predators (Borges et al. 2017b), herbivores (Borges et al. 2018a, Rego et al. 2019), saproxylic decomposers (Wetherbee et al. 2023) and fungivores (Borges et al. 2017a) and, thus, play key roles in nutrient cycling, wood decomposition and trophic regulation in Azorean forests (Borges et al. 2017b, Borges et al. 2022). A recent assessment of the conservation status of forest beetles in the Azores revealed that many species, particularly those endemic to single islands or associated with old-growth native forest, face an elevated risk of extinction due to habitat loss, fragmentation and the spread of invasive species (Borges et al. 2017). Nevertheless, for many beetle species, basic ecological information, such as local distribution and abundance, remains fragmentary or scattered amongst disparate sources, limiting their use in conservation planning.

Effective and efficient conservation of these ecologically valuable forest remnants requires robust, standardised information on species identities, distributions and origin status, ideally obtained under comparable sampling designs and covering multiple taxonomic groups. Large-scale initiatives, such as the EU NETBIOME-funded ISLANDBIODIV project (Emerson et al. 2016) and the FCT-funded MACDIV project (Malumbres-Olarte et al. 2019, Malumbres‐Olarte et al. 2021), were established precisely to generate such data, aiming to quantify diversity patterns of vascular plants, springtails, beetles and spiders across the Macaronesian islands. Within these projects, a network of sixteen 50 m × 50 m native forest plots was set up in Pico (six plots) and Terceira (ten plots) (see also Borges et al. (2025)) to investigate local and regional drivers of community assembly under a unified sampling framework. Arthropod sampling in these plots followed an optimised COBRA (Conservation Oriented Biodiversity Rapid Assessment) protocol, complemented by island-specific methods (under-bark/epiphyte and coarse woody debris/ground refugia searches) designed to improve coverage of beetle assemblages (Borges et al. 2018c).

Standardised inventories, based on the same plot network, have already been published for spiders in the native forests of Pico and Terceira (Malumbres-Olarte et al. 2019) and trees in Terceira, Tenerife and Réunion Islands (Borges et al. 2025), demonstrating the value of these data for documenting colonisation status, detecting new records and supporting biogeographical and conservation analyses. However, the beetle fauna collected in the same plots has not yet been compiled and made accessible in a comparable format using Darwin Core, the widely used biodiversity data standard for structuring and sharing occurrence records (Wieczorek et al. 2012).

## General description

### Purpose

Here, we provide the first plot-based, standardised inventory of beetles from native forests of Pico and Terceira, including abundance and colonisation status for each species. Our goal is to make these data openly available as a sample-event dataset that can be integrated with existing Macaronesian arthropod and plant datasets, thereby contributing a robust baseline for future ecological, biogeographical and long-term monitoring studies in Azorean native forests.

### Additional information

The implementation of Darwin Core Database was performed under the scope of the ongoing project BioMonI – Biodiversity monitoring of island ecosystems project under the 2022-2023 BiodivMon joint call. It was co-funded by the European Commission (GA No. 101052342).

## Project description

### Title

BioMonI-PLOTS: Standardised beetle inventory data from Pico and Terceira Islands (Azores)

### Personnel

The project was conceived by Paulo A.V. Borges, Pedro Cardoso and François Rigal.

Fieldwork: (Pico Island) - Paulo A. V. Borges, Rui Carvalho, Luis Carlos Crespo, Rosalina Gabriel, Sietze Norder, Fernando Pereira; (Terceira Island) - Isabel R. Amorim, Paulo A. V. Borges, Pedro Cardoso, Margarita Diaz, Maria Teresa Ferreira, Orlando Guerreiro, Rui Nunes, Fernando Pereira, Carla J. Rego, François Rigal.

Fieldwork permits: CCPI 18/2016/DRA; Licença 36/2016/DRA.

Parataxonomists: Alejandra Ros-Prieto.

Taxonomist: Paulo A. V. Borges.

Database management: Alejandra Ros-Prieto and Paulo A. V. Borges.

Darwin Core Database management: Paulo A. V. Borges.

### Study area description

The Azores (38°43′49″N, 27°19′10″W) are an isolated mid-Atlantic archipelago of nine volcanic islands (Fig. 1). Native vegetation across the Azores is mostly restricted to high elevations, with only ~ 5% of original habitats still protected (Gaspar et al. 2010). Lowland remnants of *Erica azorica* - *Morella faya* and *Picconia azorica* - *Morella faya* woodlands persist up to 300 m, whereas *Juniperus brevifolia* - *Ilex azorica* forests dominate between 600 and 1000 m. Former laurel forests (*Laurus azorica*) once covered over two-thirds of the islands, forming dense, hyper-humid habitats rich in bryophytes and ferns (Elias et al. 2016). Human activity, including habitat destruction and invasive species, has heavily altered natural vegetation, with the best-preserved patches found above 600 m (Gaspar et al. 2010, Elias et al. 2016).

Pico Island (436–445 km²; 0.19–0.27 Ma - million years ago) is dominated by the stratovolcano Pico Mountain (2,351 m) and features lava flows, lava tubes and volcanic pits. The Island has a temperate oceanic climate, with high humidity, persistent winds and abundant rainfall; at low elevations, mean annual temperatures are generally around 17–18°C, whereas annual precipitation is typically about 1,000–2,000 mm, increasing markedly with altitude and locally exceeding 4,000 mm in montane areas (Gil et al. 2017, Coelho et al. 2021). Terceira Island (402 km²; 0.4 Ma) is circular, formed by four volcanic complexes, with protected areas mainly in Serra de Santa Bárbara and Pico Alto. It also has a temperate oceanic climate with mild temperatures and high humidity; lowland mean annual temperatures are around 17°C, while rainfall is generally in the order of 1,500–3,000 mm per year, exceeding 3,400 mm in the highest mountain areas, where mean annual temperature may drop to about 9°C (Azevedo et al. 2004, Gaspar et al. 2010, Ávila et al. 2016). Land use in both islands follows an altitudinal gradient: urban and agricultural areas in lowlands, pastures at mid-elevations and more natural habitats in central highlands. The original native vegetation, especially mid-elevation laurel forests, has been largely replaced by exotic plantations and croplands, while subalpine scrublands (*Calluna vulgaris* and *Erica azorica*) remain in high elevations (Elias et al. 2016). Submontane laurel forests could potentially cover 49–65% of Pico, with *Juniperus brevifolia* - *Ilex azorica* montane forests up to 20% (Gaspar et al. 2010). On Terceira, native forests cover ~ 10% of the land, mostly in high-elevation central areas, while ~ 22% is legally protected (Gaspar et al. 2010).

### Design description

On Pico and Terceira Islands (Azores), we established a network of permanent 50 m × 50 m native-forest plots to quantify how community composition changes across space and forest fragmentation (Fig. 1c, d; Table 1). In total, we set up six plots on Pico and ten plots on Terceira, spanning longitudinal extents of ~ 20 km and ~ 13 km, respectively (Malumbres-Olarte et al. 2019). On Pico, plots were positioned at increasing (log-scaled) distances from a reference plot (0.1, 1, 5, 10 and 20 km), intentionally spanning the three remaining native-forest fragments. This spatial configuration was designed to test distance–decay patterns in β-diversity while maintaining a standardised sampling grain (2,500 m²) across all sites, as implemented within the MACDIV framework (Malumbres-Olarte et al. 2019, Malumbres‐Olarte et al. 2021).

On Terceira, the ten plots were randomly distributed across the Island’s four largest native-forest fragments to capture both within-fragment (α-diversity) variation and amongst-fragment (β-diversity) turnover, following the same general plot-based rationale used in earlier island-wide biodiversity assessments (Borges et al. 2018b).

All plots were placed in mid- to high-elevation native forest, structurally dominated by the endemic conifer *Juniperus brevifolia* and broadleaf evergreen trees, such as *Laurus azorica* and *Ilex azorica*, ensuring that comparisons primarily reflect spatial effects (distance, fragmentation) within a relatively consistent habitat template (Malumbres-Olarte et al. 2019, Malumbres‐Olarte et al. 2021). Fordetails on fragment sizes (on both islands; three on Pico, four on Terceira), see Gaspar et al. (2008).

### Funding

Fieldwork was supported by two projects: 1) the ERA-Net Net-Biome research framework, financed through Portuguese FCT-NETBIOME grant 0003/2011 (PB); and 2) FCT MACDIV – FCT-PTDC/BIABIC/0054/2014. CR, FR and IRA were supported by grants from Fundação da Ciência e Tecnologia – FCT-SFRH/BPD/91357/2012, FCT-PTDC/BIABIC/119255/2010 and FCT-SFRH/BPD/102804/2014, respectively. Database Darwin Core implementation were funded by the project Biodiversa+ project BioMonI – Biodiversity monitoring of island ecosystems, funded by FCT (BiodivMon/0003/2022). Open access to the general public will be financed by the AZORES BIOPORTAL (Upgrading the Azorean Biodiversity Portal Infrastructure (AZORES BIOPORTAL- PORBIOTA) to Boost Biodiversity Research, Management and Education -PORBIOTA” (DRCID, ACORES2030-FEDER-03420600).

## Sampling methods

### Study extent

We established 16 permanent 50 m × 50 m native-forest plots on Pico (6 plots) and Terceira (10 plots) (Table 1), spanning ~ 20 km and ~ 13 km longitudinal gradients, respectively. On Pico, plots were arranged at increasing (log-scaled) distances from a reference plot (0.1–20 km) across the three main forest fragments to test distance–decay in β-diversity, whereas, on Terceira, plots were randomly distributed across four forest fragments to evaluate both α- and β-diversity patterns (Malumbres-Olarte et al. 2019, Malumbres‐Olarte et al. 2021).

### Sampling description

**Figure 2.**
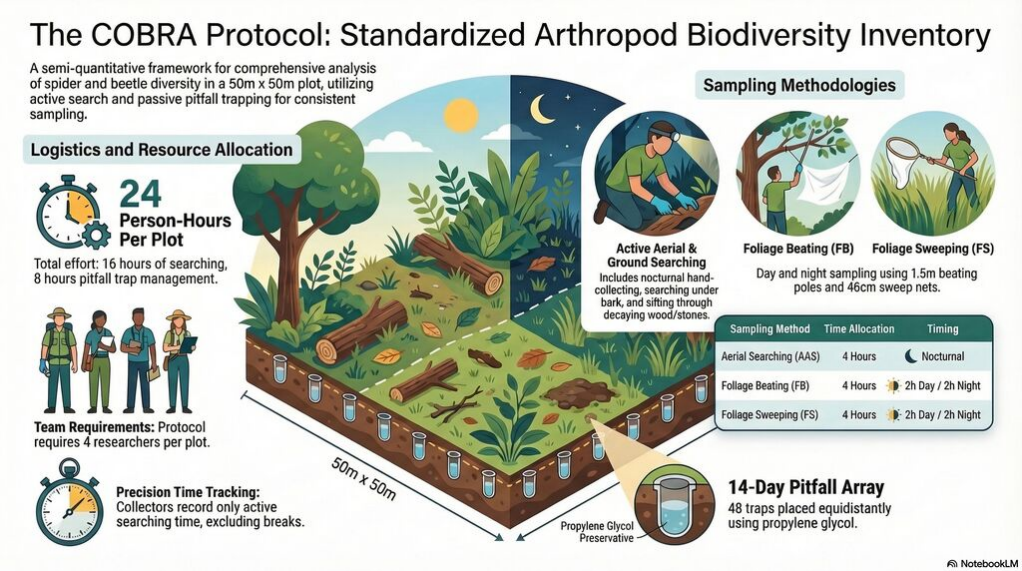
The COBRA Protocol Infographics (performed using NotebookLM).

We sampled all plots using the optimised and standardised COBRA protocol (Conservation Oriented Biodiversity Rapid Assessment), designed to maximise species recovery per unit effort, while producing fully comparable samples amongst plots and islands (Borges et al. 2018c). Beetles were sampled with the modified COBRA protocol in 50 m × 50 m plots. The full inventory module comprised (see also Fig. 2): (i) nocturnal active aerial searching (AAS; four 1-h samples), in which collectors searched by hand, forceps, pooter or brush for specimens found above knee level and transferred them directly to vials with alcohol; (ii) foliage sweeping with a round sweep net (46 cm opening diameter) in bushes and tall herbs during the day (FSD; two 1-h samples) and at night (FSN; two 1-h samples); (iii) foliage beating during the day (FBD; two 1-h samples) and at night (FBN; two 1-h samples), using a 110 cm × 80 cm framed white beating tray as a drop-cloth and a wooden pole at least 1.5 m long to strike branches as high as possible, with effective sampling time including beating, searching the tray for fallen arthropods and transferring them to vials; and (iv) pitfall trapping (PIT), using 48 pitfalls placed immediately outside the plot perimeter, with 12 traps along each side of the square plot at equal spacing. Pitfalls consisted of 33 cl plastic cups ca. 8 cm wide at the top and ca. 12 cm deep, filled with 3–4 cm of 100% propylene glycol and protected by plastic plates held ca. 2 cm above the soil surface; traps remained active for 14 days and each group of four contiguous traps was pooled as one sample, yielding 12 pitfall samples per plot. To improve representation of cryptic and microhabitat-specialised taxa, the protocol also included two diurnal targeted searches: under-bark/epiphyte searches (ABS; two 1-h samples), aimed at specimens above ground level under bark, lichens and bryophytes, with debris checked on a beating tray; and ground wood/stone searches (GWS; two 1-h samples), aimed at specimens below knee level in decaying trunks, dead wood on the ground and beneath stones, with debris searched on a 1 m² white cloth sheet. All active methods were time-standardised and only effective collecting time was counted (Borges et al. 2018c). Sampling was conducted in July 2016 (between 3 and 26) in Pico Island during two expeditions (MACDIV Project) and between June and September 2012 in Terceira Island (ISLANDBIODIV Project). Since the team is based on Terceira Island, sampling occurred in a wide timeline.

Additionally, litter/soil (LIT) samples were available for the Terceira plots from supplementary sampling targeted at soil Collembola (Cicconardi et al. 2017). In each of the 10 Terceira sites, 10 randomly placed cylindrical soil cores (8 cm diameter, ca. 10 cm depth, depending on soil depth) were collected, with a minimum spacing of 5 m between cores. After pooling the cores to obtain 4–5 litres of soil, a 3.6 litre subsample was divided amongst three Tullgren funnels and arthropods were extracted into ethanol over 14 days using a 40-W incandescent light source (Cicconardi et al. 2017).

### Step description

A reference collection of Azorean arthropods, housed at the Dalberto Teixeira Pombo Insect Collection (University of the Azores), was used to help specimen identification. The taxonomic nomenclature and colonisation status of the species follows the most recent checklist of Azorean arthropods (Borges et al. 2022).

## Geographic coverage

### Description

Pico and Terceira Islands, the Azores, Macaronesia, Portugal.

### Coordinates

38.438 and 38.762 Latitude; -28.423 and -27.198 Longitude.

## Taxonomic coverage

### Description

The following families of Coleoptera are covered: Apionidae, Carabidae, Ciidae, Cryptophagidae, Curculionidae, Dytiscidae, Elateridae, Hydrophilidae, Leiodidae, Nitidulidae, Phalacridae, Ptiliidae, Ptinidae, Scraptiidae, Staphylinidae and Zopheridae.

## Traits coverage

Traits for the studied species can be consulted in Oyarzabal et al. (2026).

## Temporal coverage

**Data range:** 2012-6-08 – 2016-7-26.

## Collection data

### Collection name

Entomoteca Dalberto Teixeira Pombo at University of Azores.

### Collection identifier

DTP

### Specimen preservation method

All specimens were preserved in 96% ethanol.

### Curatorial unit

Dalberto Teixeira Pombo insect collection at the University of the Azores (Curator: Paulo A. V. Borges).

## Usage licence

### Usage licence

Creative Commons Public Domain Waiver (CC-Zero)

## Data resources

### Data package title

BioMonI-PLOTS: Standardised beetle inventory data from Pico and Terceira Islands (Azores)

### Resource link

https://doi.org/10.15468/fcebeu

### Alternative identifiers

https://www.gbif.org/dataset/bd42f57d-c7c2-4b59-a12c-550e01b2ebcc

### Number of data sets

2

### Data set 1.

#### Data set name

Event Table

#### Data format

Darwin Core Archive format

#### Character set

UTF-8

#### Download URL

https://ipt.gbif.pt/ipt/resource?r=biomoni_beetles

#### Data format version

1.2

#### Description

The dataset was published in the Global Biodiversity Information Facility platform, GBIF (Borges 2026). The following data table includes all the records for which a taxonomic identification of the species was possible. The dataset submitted to GBIF is structured as a sample event dataset that has been published as a Darwin Core Archive (DwCA), which is a standardised format for sharing biodiversity data as a set of one or more data tables. The core data file contains 308 records (eventID). This GBIF IPT (Integrated Publishing Toolkit, Version 2.5.6) archives the data and, thus, serves as the data repository. The data and resource metadata are available for download in the Portuguese GBIF Portal IPT.

**Data set 1.** 

Column label	Column description
locationID	Identifier of the location.
minimumElevationInMetres	The lower limit of the range of elevation (altitude, usually above sea level), in metres.
decimalLatitude	Approximate centre point decimal latitude of the field site in GPS coordinates.
decimalLongitude	Approximate centre point decimal longitude of the field site in GPS coordinates.
eventID	Identifier of the events, unique for the dataset.
samplingProtocol	The sampling protocol used to capture the species.
fieldNumber	An identifier given to the event in the field. Often serves as a link between field notes and the Event.
sampleSizeValue	The numeric amount of time spent in each sampling.
sampleSizeUnit	The unit of the sample size value.
samplingEffort	The amount of time of each sampling.
eventDate	Date or date range the record was collected.
year	Year of the event.
month	Month of the event.
day	Day of the event.
habitat	The surveyed habitat.
islandGroup	Name of archipelago.
island	Name of the island.
country	Country of the sampling site.
countryCode	ISO two-letter code of the country.
stateProvince	Name of the province.
municipality	Municipality of the sampling site.
locality	Name of the locality.
locationRemarks	Details on the locality site.
geodeticDatum	The ellipsoid, geodetic datum or spatial reference system (SRS), upon which the geographic coordinates given in decimalLatitude and decimalLongitude are based.
coordinateUncertaintyInMetres	Uncertainty of the coordinates of the centre of the sampling plot.
coordinatePrecision	Precision of the coordinates.
georeferenceSources	A list (concatenated and separated) of maps, gazetteers or other resources used to georeference the Location, described specifically enough to allow anyone in the future to use the same resources.
datasetName	Name of the dataset.

### Data set 2.

#### Data set name

Occurrence table

#### Data format

Darwin Core Archive format

#### Character set

UTF-8

#### Download URL

https://ipt.gbif.pt/ipt/resource?r=biomoni_beetles

#### Data format version

1.2

#### Description

The dataset was published in the Global Biodiversity Information Facility platform, GBIF (Borges 2026). The following data table includes all the records for which a taxonomic identification of the species was possible. The dataset submitted to GBIF is structured as an occurrence table that has been published as a Darwin Core Archive (DwCA), which is a standardised format for sharing biodiversity data as a set of one or more data tables. The core data file contains 539 records (occurrenceID). This GBIF IPT (Integrated Publishing Toolkit, Version 2.5.6) archives the data and, thus, serves as the data repository. The data and resource metadata are available for download in the Portuguese GBIF Portal IPT.

**Data set 2.** 

Column label	Column description
eventID	Identifier of the events, unique for the dataset.
type	Type of the record, as defined by the Dublin Core Standard.
licence	Reference to the licence under which the record is published.
institutionID	Reference to the licence under which the record is published.
collectionID	The identity of the collection publishing the data.
institutionCode	The code of the institution publishing the data.
collectionCode	The code of the collection where the specimens are conserved (DTP).
basisOfRecord	The nature of the data record.
occurrenceID	Identifier of the record, coded as a global unique identifier.
recordedBy	Name of the person who performed the sampling of the specimens.
identificationRemarks	Information about morphospecies identification (code in Dalberto Teixeira Pombo Arthropod Collection).
scientificName	Complete scientific name including author and year.
taxonRank	Lowest taxonomic rank of the record.
kingdom	Kingdom name.
phylum	Phylum name.
class	Class name.
order	Order name.
family	Family name.
genus	Genus name.
specificEpithet	Specific epithet.
infraspecificEpithet	Infrapecific epithet.
scientificNameAuthorship	Name of the author of the lowest taxon rank included in the record.
establishmentMeans	The process of establishment of the species in the location, using a controlled vocabulary: 'native', 'introduced', 'endemic', "uncertain".
organismQuantity	A number or enumeration value for the quantity of organisms.
organismQuantityType	The type of quantification system used for the quantity of organisms.
identifiedBy	A list (concatenated and separated) of names of people, groups or organisations who assigned the Taxon to the subject.
dateIdentified	The date on which the subject was determined as representing the taxon.

## Additional information

We recorded a total of 43 beetle morphospecies, belonging to 16 families, from native forest habitats on Pico and Terceira Islands, with an overall abundance of 1,787 individuals. From those, 39 taxa were identified at species or subspecies levels (Table 2). On Pico Island, we recorded 25 taxa (23 identified at species and subspecies) belonging to 13 families, for a total of 620 individuals. On Terceira Island, 30 taxa were detected (28 identified at species and subspecies), distributed across 10 families, with an overall abundance of 1,167 individuals (Table 2). Considering the number of Azorean endemic beetle species known to occur in native forests, our sampling recovered a substantial proportion of the native forest-specialist fauna on both islands: 10 of the 22 species known from Pico Island (45.5%) and 11 of the 18 species known from Terceira Island (61.1%) (Borges et al. 2017a, Borges et al. 2017b). These values indicate that the survey captured nearly half of the endemic forest-specialist beetle fauna of Pico and more than half of that of Terceira, supporting the relevance of the dataset for characterising native forest beetle assemblages on both islands.

Most taxa were identified to species or subspecies level (39 out of 43), allowing a detailed assessment of their colonisation status. The assemblage was clearly dominated by endemic species, both in terms of richness and abundance, followed by introduced taxa. A considerable number of species had an indeterminate status, exceeding the number of native non-endemic taxa.

Overall, a total of 16 endemic species were recorded across both islands (10 on Pico and 11 on Terceira). Introduced species accounted for 10 taxa (seven on Pico and seven on Terceira), while nine species had an uncertain colonisation status (four on Pico and seven on Terceira). Native species were represented by four taxa (two on Pico and three on Terceira). In addition, four taxa could not be identified to the species level (two on Pico and two on Terceira).

**Figure 3.**
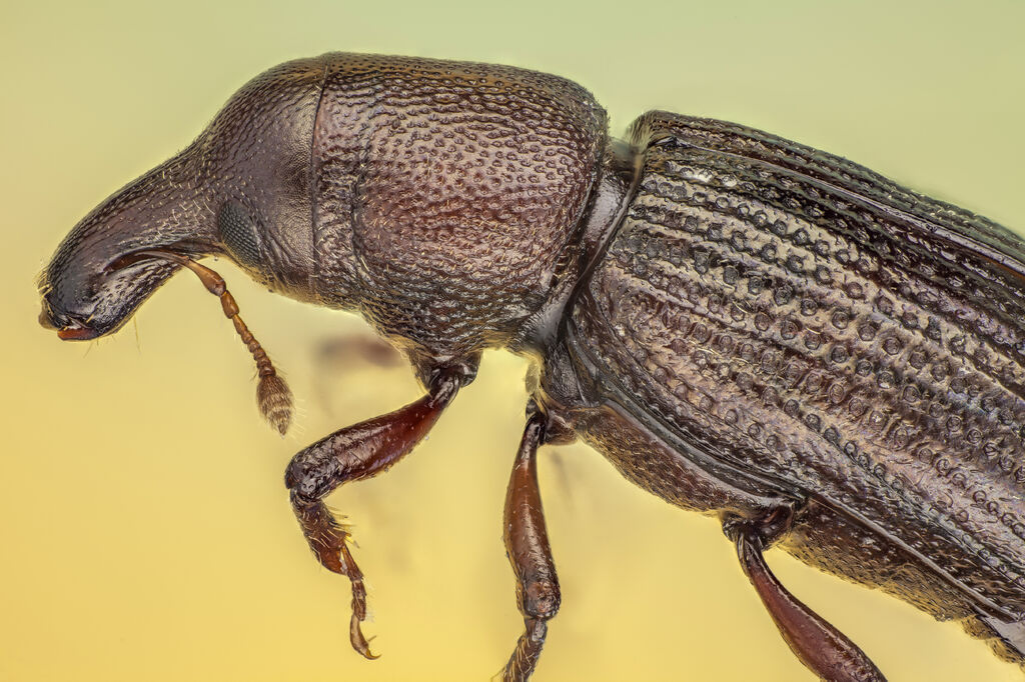
*Pseudophloeophagus tenax borgesi* (Stüben, 2022) (Credit: Javier Torrent, Azorean Biodiversity Group).

Endemic beetles accounted for the majority of individuals collected and were particularly well represented within Curculionidae, Carabidae, Staphylinidae and Zopheridae. Several endemic species were highly abundant and largely associated with native forest habitats, notably:

*Pseudophloeophagus tenax borgesi* (Stüben, 2022) (Fig. 3) is a recently described subspecies, likely distributed across all the Azorean islands. It primarily inhabits under-bark and deadwood microhabitats, while the nominotypic taxon occurs on Madeira.

**Figure 4.**
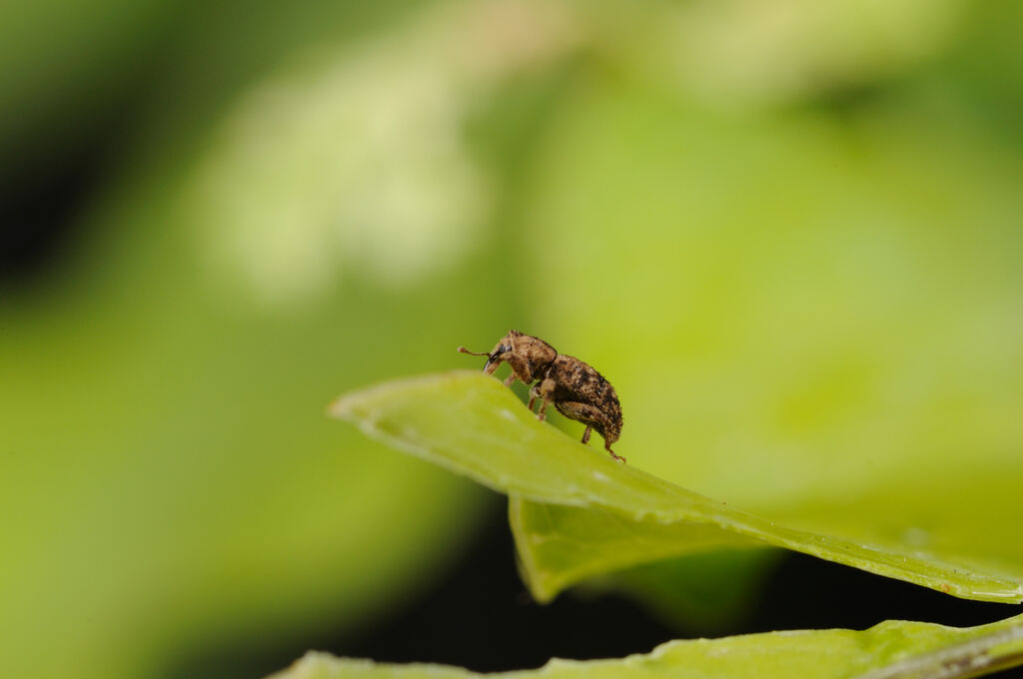
*Calacalles subcarinatus* (Israelson, 1984) (credit: Paulo A. V. Borges).

*Calacalles subcarinatus* (Israelson, 1984) (Fig. 4) is a widespread and abundant species occurring on all islands at altitudes between 100 and 1200 m. It mainly inhabits native forests dominated by endemic vegetation, but also colonises exotic plantations and agricultural areas. Both adults and larvae are herbivorous, feeding on plant tissues throughout the day and night, with seasonal peaks in spring and summer.

**Figure 5.**
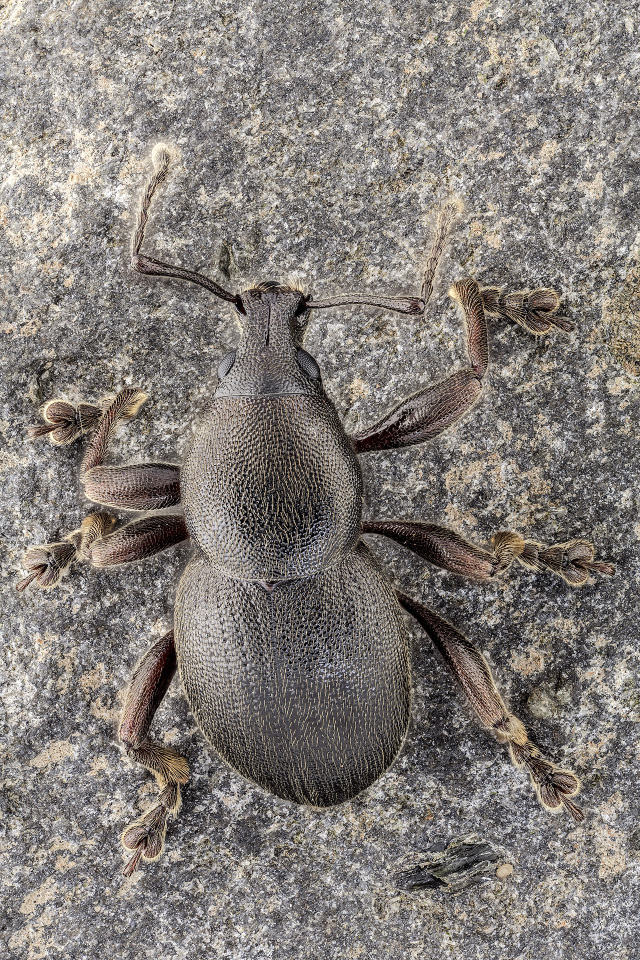
*Drouetius borgesi borgesi* (Machado, 2009) (credit: Javier Torrent, Azorean Biodiversity Group).

*Drouetius borgesi borgesi* (Machado, 2009) (Fig. 5) is a small, endemic true weevil associated with native forest ecosystems of the Azores (Borges et al. 2017b). The species is herbivorous, feeding predominantly on the foliar tissues of native forest plants and occurs across an altitudinal range of approximately 300 to 1100 m above mean sea level (a.s.l.) (Borges et al. 2017b). Under the IUCN Green Status of Species framework, *D. borgesi* is currently assessed as Largely Depleted (Borges and Oyarzabal 2025b). This status reflects ongoing threats related to habitat degradation and loss, largely driven by invasive plant species that alter soil cover and forest structure, as well as projected impacts of climate change, including increased drought frequency.

*Anaspis proteus* (Wollaston, 1854) is endemic to the Canary Islands, Madeira and the Azores. It is a common and highly adaptable species, occurring from coastal areas to mountain summits. Adults are associated with a wide variety of plants, especially Asteraceae and Apiaceae, while larvae develop in decomposing wood, contributing to nutrient recycling in forest ecosystems.

*Tarphius furtadoi* (Borges & Serrano, 2017) belongs to the *tornvalli* complex and is a nocturnal, fungivorous, univoltine species (Borges et al. 2017a). It inhabits soil and subcortical layers of dead wood in well-preserved native forests on São Jorge, Pico and Faial, at elevations between 250 and 1000 m. Although locally abundant, populations are declining due to native forest loss, plantation management and the spread of invasive plants (Borges et al. 2017b).

*Calathus carvalhoi* (Serrano & Borges, 1986) is a very rare ground-beetle that was originally found in a low elevation site at Terceira island (Terra-Chã) dominated by exotic trees in the year of 1983 and, in the current study, is for the first time recorded in Terra-Brava Pristine native forest in the year of 2012. Further specimens were also sampled in this same Plot (Terra-Brava) in pitfall traps in 2020 (Lhoumeau et al. 2024) and 2021 (Pozsgai et al. 2024).

**Figure 6.**
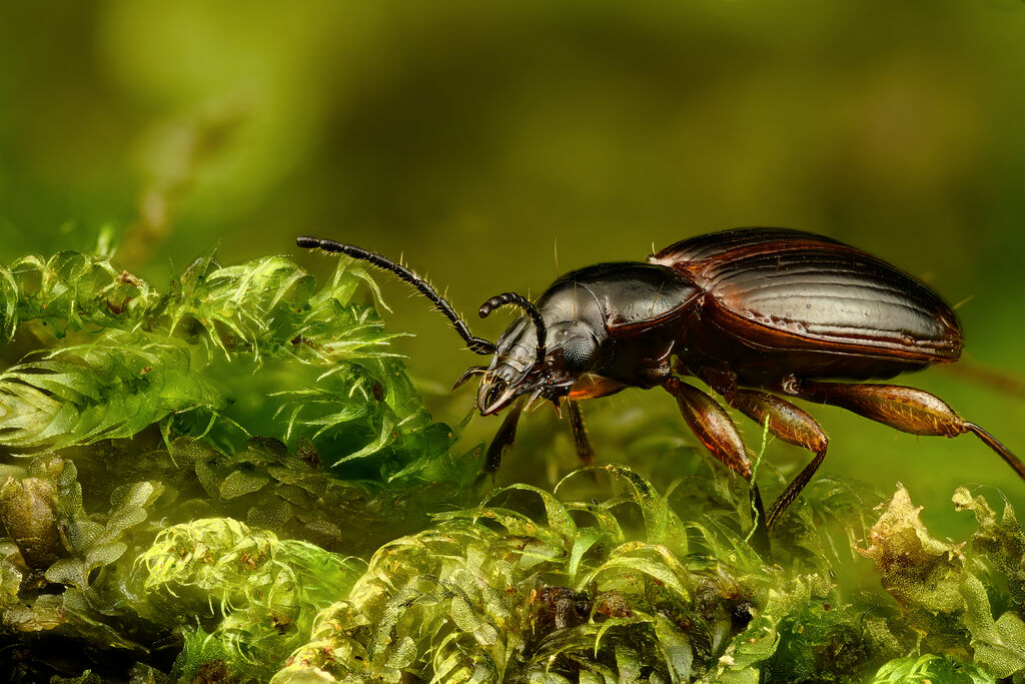
*Trechus terrabravensis* (Borges, Serrano & Amorim, 2004) (credit: Javier Torrent, LIFE BEETLES).

*Trechus terrabravensis* (Borges, Serrano & Amorim, 2004) (Fig. 6) is a small, flightless ground beetle endemic to Terceira Island (Borges et al. 2022). The species is strictly terrestrial and predatory, inhabiting humid native laurel forests dominated by *Laurus azorica*, *Juniperus azorica* and *Ilex perado* (Borges et al. 2004). It occurs primarily within dense moss and fern litter at elevations ranging from approximately 500 to 1000 m a.s.l. (Borges et al. 2004). According to the IUCN Green Status of Species (Borges 2022), *T. terrabravensis* is assessed as Slightly Depleted, indicating that, despite its restricted distribution, the species persists in relatively intact native forest remnants.

**Figure 7.**
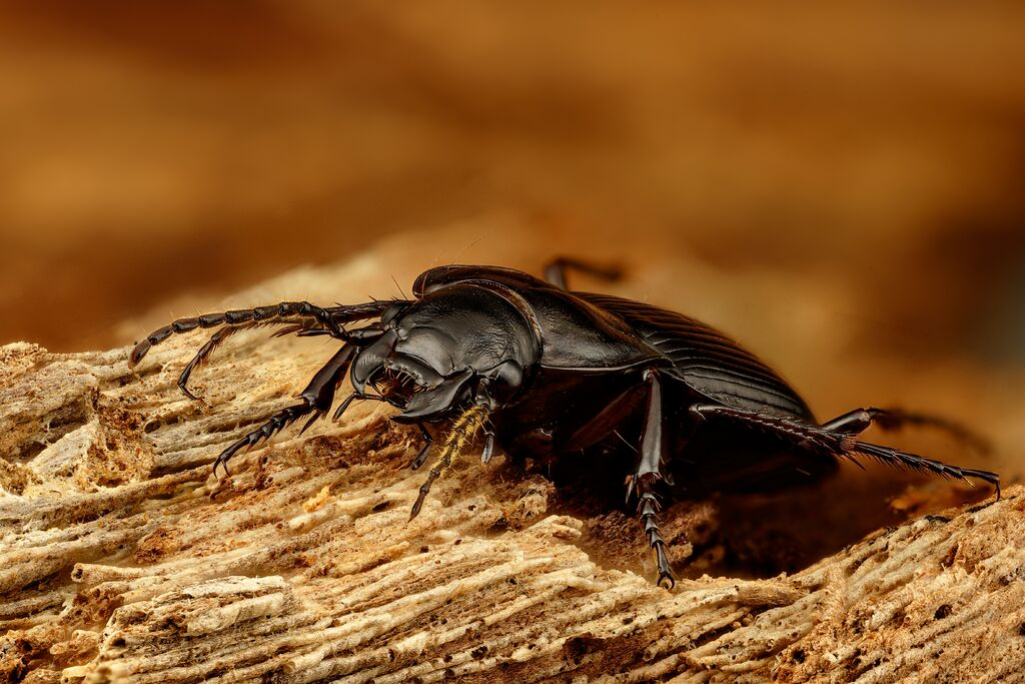
*Cedrorum azoricus azoricus* (Borges & Serrano, 1993) (credit: Javier Torrent, Azorean Biodiversity Group).

*Cedrorum azoricus* (Borges & Serrano, 1993) (Fig. 7), is a ground beetle restricted to native forest habitats of Terceira, Pico and Santa Maria Islands (Borges et al. 2022), with one subspecies restricted to Pico Island (*C. a. caveirensis*) and another restricted to Santa Maria and Terceira Islands (*C. a. azoricus*) (Borges and Serrano 1993). The species exhibits morphological traits adapted to a forest-floor lifestyle, including a robust body form and well-developed legs and mandibles that facilitate active foraging and interaction with the substrate (Borges and Serrano 1993). Its Green Status assessment classifies the species as Largely Depleted (Borges and Oyarzabal 2025a), reflecting the fragmented distribution of populations across isolated native forest remnants. There is clear evidence of a continuing decline in both occupancy and habitat quality, primarily driven by habitat alteration and the spread of invasive plant species that degrade forest structure and microhabitat conditions.

**Figure 8.**
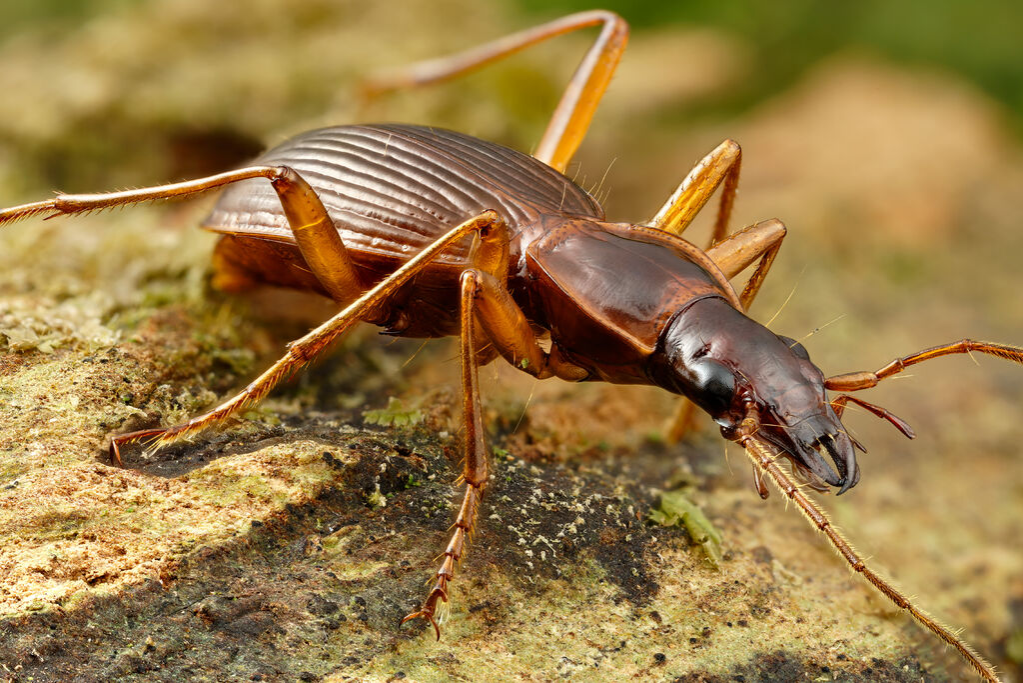
*Pseudanchomenus aptinoides* (Tarnier, 1860) (credit: Javier Torrent, LIFE BEETLES).

*Pseudanchomenus aptinoides* (Tarnier, 1860) (common name: Laurocho) (Fig. 8) is a large predator living in the pristine forests of Pico Island. The Laurocho is currently Largely Depleted, with a Species Recovery Score of 42%, because it has been permanently extirpated from São Miguel Island and survives only in Pico Island (Borges 2024). On Pico, three subpopulations remain: one is present, but non-viable and two are viable, but non-functional. Ongoing habitat actions, especially cattle exclusion and invasive plant control supported by LIFE BEETLES and LIFE IP AZORES NATURA, are preventing further degradation; without them, none of the subpopulations would currently be viable (a Medium Conservation Legacy). The species is also conservation dependent: if these measures stopped now, all subpopulations are expected to become non-viable within ~ 10 years (Medium Conservation Dependence). With continued management and improving habitat quality, all extant subpopulations are projected to reach viable status by 2033 (a Low Conservation Gain) (Borges 2024). However, over the next century, climate-driven drying is expected to progressively reduce habitat suitability, lowering the Recovery Score from 42% to 25%, indicating a Negative Recovery Potential (Borges 2024).

The recent IUCN Green Status of Species assessments add a crucial “recovery lens” to the already-available Red List evidence for Azorean endemics (Borges et al. 2017b, Borges et al. 2018a), by quantifying how close each species is to being ecologically functional across its indigenous range and by estimating the impact of past, ongoing and future conservation through standardised metrics (Species Recovery Score plus Conservation Legacy/Dependence/Gain) (Grace et al. 2021). This is especially relevant on oceanic islands, where many invertebrates persist in small habitat remnants and extinction risk alone can hide severe functional depletion long before global extinction becomes imminent (Triantis et al. 2010).

For *Trechus terrabravensis*, the Slightly Depleted classification (Species Recovery Score ~83%) indicates that, despite a very narrow range and flightlessness, the species still persists in comparatively intact Terceira native forest, implying that strict protection of key laurel forest remnants can maintain near-functional populations for some specialised predators (Borges 2022). In contrast, *Cedrorum azoricus* and *Drouetius borgesi*, being Largely Depleted, signal substantial loss of ecological functionality and/or spatial representation across their indigenous ranges, consistent with fragmented occupancy, declining habitat quality and strong pressure from invasive plants (and, for *D. borgesi*, plausible future sensitivity to drying and climate-driven microhabitat change) (Borges and Oyarzabal 2025a, Borges and Oyarzabal 2025b).

From a management and policy perspective, the Green Status results provide (Grace et al. 2021): (i) a quantitative baseline against which future re-assessments can measure real recovery; (ii) a way to prioritise actions that increase the Species Recovery Score (e.g. invasive plant control, restoration of humid forest-floor structure and safeguarding microclimatic refugia) and (iii) an evidence framework to communicate the benefits and necessity of continued investment, because “Largely Depleted” species are often expected to show high dependence on sustained conservation even when extinction risk is being contained. Within the IUCN Portals framework, the delivery of these gains will depend on the maintainance of standardised and repeatable monitoring approaches (e.g. permanent plot networks and optimised protocols for arthropod sampling) that can detect occupancy and abundance shifts through time and tie them to specific interventions.

## Figures and Tables

**Figure 1a.**
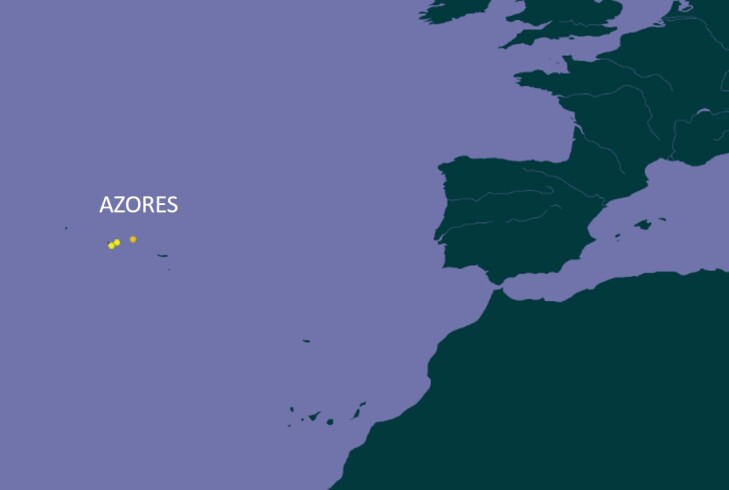
The location of Azores in Atlantic Ocean;

**Figure 1b.**
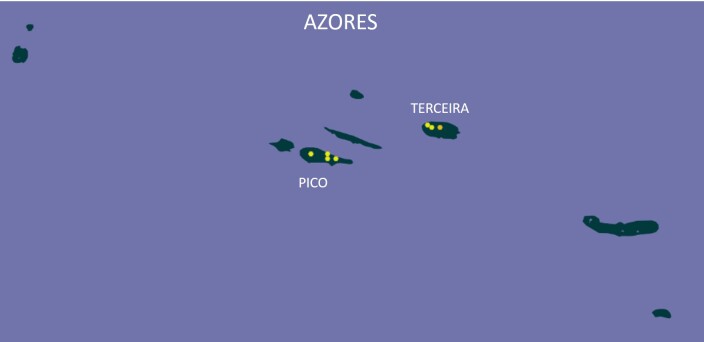
The two studied islands in Azores;

**Figure 1c.**
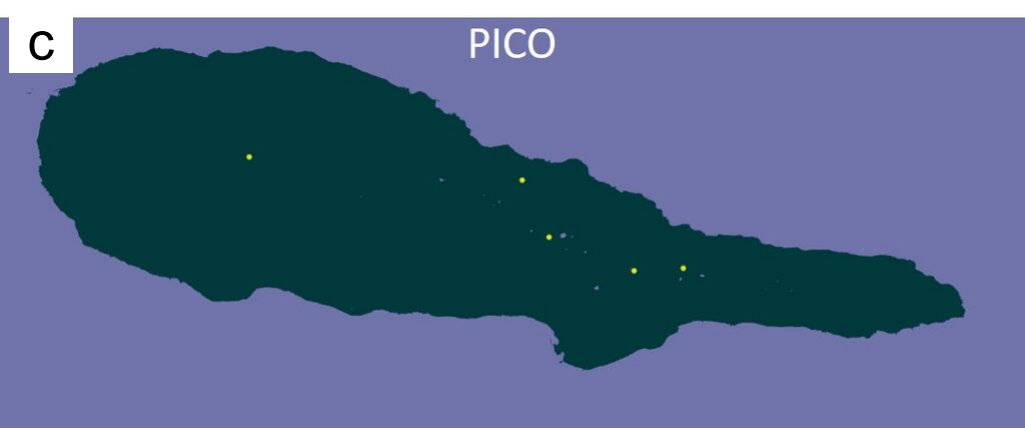
The location of plots in Pico Island. The easternmost point corresponds to two plots that are only 100 m apart;

**Figure 1d.**
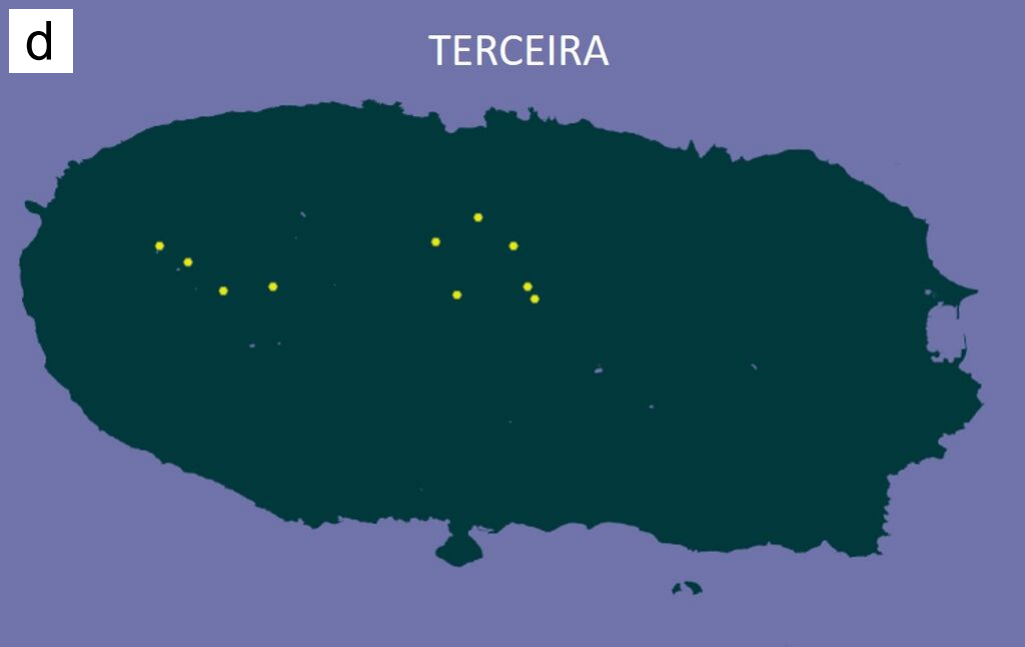
The location of plots in Terceira Island.

**Table 1.** Beetle sampling plots in native forests of Pico and Terceira Islands (Azores), surveyed using a standardised plot-based protocol. The table includes plot codes, minimum elevation, latitude and longitude, providing a baseline sample-event dataset for ecological, biogeographical and long-term monitoring studies in Azorean native forests.

Plot Code	Min Elevation	Latitude	Longitude
PIC-NFCA-T-09	935	38.4377	-28.2107
PIC-NFCA-T-22	960	38.438	-28.1843
PIC-NFCA-T-24	915	38.4378	-28.2106
PIC-NFLC-T-02	816	38.4561	-28.2577
PIC-NFMP-T-03	470	38.4877	-28.2733
PIC-NFPR-T-23	800	38.4999	-28.4229
TER-NFBF-T-01	694	38.7618	-27.2193
TER-NFBF-T-02	575	38.7521	-27.2331
TER-NFBF-TP41	686	38.7502	-27.2072
TER-NFPG-T-33	651	38.7334	-27.2271
TER-NFSB-T-07	693	38.7372	-27.2899
TER-NFSB-T164	890	38.7355	-27.3074
TER-NFSB-TE48	748	38.7521	-27.3313
TER-NFSB-TE49	930	38.7471	-27.3196
TER-NFTB-T-15	639	38.7364	-27.2006
TER-NFTB-T-18	668	38.7323	-27.1981

**Table 2.** List of sampled taxa in Pico and Terceira Islands.

**Family**	**Scientific Name**	**Colonisation Status**	**PICO**	**TERCEIRA**	**Total**
Apionidae	*Aspidapion radiolus* (Marsham, 1802)	introduced	1		1
Carabidae	*Anisodactylus binotatus* (Fabricius, 1787)	introduced		3	3
	*Calathus carvalhoi* (Serrano & Borges, 1986)	endemic		2	2
	*Cedrorum azoricus azoricus* (Borges & Serrano, 1993)	endemic		71	71
	*Cedrorum azoricus caveirensis* (Borges & Serrano, 1993)	endemic	2		2
	*Ocys harpaloides* (Audinet-Serville, 1821)	native	3		3
	*Paranchus albipes* (Fabricius, 1796)	introduced	42	282	324
	*Pseudanchomenus aptinoides* (Tarnier, 1860)	endemic	9		9
	*Stenolophus teutonus (Schrank, 1781)*	native		2	2
	*Trechus terrabravensis* (Borges, Serrano & Amorim, 2004)	endemic		79	79
Ciidae	*Atlantocis gillerforsi* (Israelson, 1985)	endemic	1	3	4
Cryptophagidae	*Cryptophagus* sp.			1	1
Curculionidae	*Calacalles subcarinatus* (Israelson, 1984)	endemic	95	105	200
	Curculionidae		4		4
	*Drouetius borgesi borgesi* (Machado, 2009)	endemic		102	102
	*Otiorhynchus rugosostriatus* (Goeze, 1777)	introduced	5	1	6
	*Phloeosinus gillerforsi* (Bright, 1987)	endemic		1	1
	*Pseudechinosoma nodosum* (Hustache, 1936)	endemic	21	4	25
	*Pseudophloeophagus tenax borgesi* (Stüben, 2022)	endemic	195	270	465
	*Rhopalomesites tardyi* (Curtis, 1825)	introduced	1	3	4
	*Sitona discoideus* (Gyllenhal, 1834)	introduced		1	1
Dytiscidae	*Agabus godmanni* (Crotch, 1867)	endemic	2		2
Elateridae	*Alestrus dolosus* (Crotch, 1867)	endemic	4	78	82
Hydrophilidae	*Cercyon haemorrhoidalis* (Fabricius, 1775)	introduced		7	7
Leiodidae	*Catops coracinus* (Kellner, 1846)	native		3	3
Nitidulidae	*Stelidota geminata* (Say, 1825)	introduced	1		1
Phalacridae	Phalacridae		2		2
Ptiliidae	*Ptenidium pusillum* (Gyllenhal, 1808)	introduced	1	5	6
Ptinidae	*Anobium punctatum* (De Geer, 1774)	introduced	1		1
Scraptiidae	*Anaspis proteus* (Wollaston, 1854)	native	139	51	190
Staphylinidae	*Aleochara bipustulata* (Linnaeus, 1760)	uncertain		1	1
	*Atheta* sp.			1	1
	*Atheta aeneicollis* (Sharp, 1869)	uncertain		1	1
	*Atheta fungi* (Gravenhorst, 1806)	uncertain	5		5
	*Carpelimus corticinus* (Gravenhorst, 1806)	uncertain	2	4	6
	*Euconnus azoricus* (Franz, 1969)	endemic	1		1
	*Notothecta dryochares* (Israelson, 1985)	endemic		16	16
	*Ocypus aethiops* (Waltl, 1835)	uncertain		34	34
	*Ocypus olens* (Müller, 1764)	uncertain	2		2
	*Proteinus atomarius* (Erichson, 1840)	uncertain	10	25	35
	*Quedius curtipennis* (Bernhauer, 1908)	uncertain		10	10
	*Xantholinus longiventris* (Heer, 1839)	uncertain		1	1
Zopheridae	*Tarphius furtadoi* (Borges & Serrano, 2017)	endemic	71		71
**Grand Total**			**620**	**1167**	**1787**
